# Research on Current Drive System of Magnetorheological Damper Based on Fuzzy PI Control

**DOI:** 10.3390/ma15248893

**Published:** 2022-12-13

**Authors:** Wei Li, Huijun Liang, Dongbin Xia, Jie Fu, Lei Luo, Miao Yu

**Affiliations:** Key Lab for Optoelectronic Technology and Systems, Ministry of Education, College of Optoelectronic Engineering, Chongqing University, Chongqing 400044, China

**Keywords:** MR damper, BUCK, Fuzzy-PI, current driver

## Abstract

Magnetorheological dampers (MRD) are increasingly used in smart structural damping systems due to their good damping properties. In practical applications, as a nonlinear device, the parameters of the internal excitation coil of the magnetorheological damper will change during operation under the influence of the temperature and external environment, deteriorating the dynamic performance of the output current of the driver and reducing the damping effect of the system. Therefore, the current driver needs to be optimized for this phenomenon in order to ensure accurate current output. In this paper, a mathematical model of the buck circuit combined with the MRD equivalent circuit is established, and after analyzing the model, the parameters of the PI controller are rectified to lay the foundation for the design of the adaptive law. Then, with the help of the fuzzy control method, a fuzzy PI control strategy for MRD current driver is established, which enables the current driving system to adjust the control parameters adaptively when the MRD parameters change and ensure the accurate driving current output. The experimental results demonstrate that the fuzzy PI control strategy has a stronger robustness in the face of parameter changes of the control object compared with the traditional PI control at a system parameter change rate of 40%.

## 1. Introduction

The MRD is an intelligent controllable device developed based on the magnetorheological properties of magnetorheological materials [1], and its damping force value can be adjusted by the excitation current [2]. It has the advantages of simple structure, fast response [3], low energy consumption, and large dynamic range [4]. It has great promise for semi-active vibration control [5], such as intelligent suspension damping for automobiles [6], and bridge and building damping [7]. Affected by the internal parameters of the damper and other factors, the dynamic response has a time delay and affects the control accuracy, and the damping performance of the system cannot be guaranteed [8]. After analyzing the dynamic characteristics of the MRD, it can be observed that the link from the driver drive voltage to the damper current has a great influence on the dynamic characteristics of the damper [9]. In order to ensure the dynamic performance of the damper, the current driver of the MRD needs to have a high response rate. As the excitation coil inside the MRD is affected by temperature and external environment during operation, its internal resistance will change within a certain range [10]. If the driver cannot adaptively respond to the parameter changes of the MRD, the drive current will be overshot, as well as other consequences, affecting the precise accuracy of the MRD damping force output and causing the deterioration of the damping effect. Therefore, it is of high research value to develop a current driver with an adaptive capability to promote the dynamic performance of the MRD, and ensure the accurate adjustment of the damping force to improve the damping effect of the system.

In the design of the current drive circuit structure, because the buck converter [11] has the advantages of a simple circuit structure, fast response speed and high conversion efficiency, it is widely used in the current drive of MRDs [12]. In order to ensure a stable current response, the drive circuit usually needs to introduce a control strategy. Therefore, researchers employed PI [13], Sliding Mode Control (SMC) [14], Active Disturbance Rejection Control (ADRC) [15], etc. to the current drive control system. Zhu H [16] used the adaptive ADRC in the design of the current driver of the MRD, which discarded the high dependence of the system on the model accuracy. It is verified by experiments that the controller improves the parameter adaptability of the closed-loop system. Lu, H [17] proposes adaptive PI control to improve the response of MRD current driver, and the designed controller is experimentally verified to have good real-time performance and stability, but it did not analyze the parameter uncertainty of MRD and the overshoot control of the drive current for suppression. Ha. Q. P [18] established a current-dependent algebraic model of the hysteresis in the force-velocity relationship of the damper and used a sliding film control algorithm to control the excitation current of the MRD, and the experimental results demonstrated the effectiveness of the proposed current-driven scheme for the control of the MRD. By analyzing the references, the comparison between different controllers is shown in Table 1. The traditional PI control has a simple structure, high reliability, and convenient parameter setting, and is widely used in industrial field control [19]. Facing the use environment with a high rate of change of the current driver and complex signal tracking, the PI control controller can achieve a faster response speed [20]. However, since the MRD is a nonlinear device, the accuracy of the PI controller will decrease or even oscillate when the damper parameters change or are disturbed by the outside under the fixed PI parameters. The sliding mode control can overcome the uncertainty of the system and has a strong robustness to unknown disturbances [21]. However, when the state trajectory reaches the sliding mode surface, the inertia of the system makes it shuttle back and forth on both sides of the sliding mode surface, resulting in jitter [22], and the precision driving response cannot be guaranteed in the application environment where the MRD requires a fast current. The ADRC controller [23] has less dependence on the mathematical model of the system and estimates the system disturbance through the extended state observer; thus, it has a strong adaptability to the uncertainty of the system. This is because the ADRC controller has many parameters in the application and the adjustment is complicated; the real-time performance of the system is reduced due to the increase in the amount of calculations. The fuzzy PI controller [24] uses fuzzy rules and system response to perform fuzzy reasoning to achieve the optimal adjustment of the PI parameters. It has the advantages of simple structure, small amount of calculation, and easy parameter tuning [25]. It has great advantages in the application environment, where the magnetorheological damping system emphasizes real-time performance and stability. Wang, H [26] designed a neuro-fuzzy recognition system to address the problem that the strong nonlinearity of MRD causes difficulties in the construction of its inverse model, and the simulation verified that the proposed neuro-fuzzy system can identify the inverse model of MRD accurately. Rashid, M. M [27] designed a hybrid fuzzy controller based on quarter suspension, and demonstrated through experiments that the designed hybrid fuzzy controller can effectively suppress random road disturbances and improve the smoothness of the vehicle. Deng, Z [28] designed a fuzzy PID control controller with the vehicle’s vertical and pitch acceleration as the control target for the vibration reduction requirements of the vehicle magneto-rheological suspension system, and the simulation demonstrated that the designed controller can effectively improve the vehicle attitude.

From the above studies, it can be observed that MRD devices and fuzzy PI control algorithms are now widely used in the field of intelligent vibration damping. However, most scholars mainly focus on the mechanical model construction and suspension system control aspects of MRD, and treat the current driver as an ideal device or use only the PI controller with fixed parameters in the research process, while lacking the model analysis of the driving circuit, whose controller parameters are time-consuming and complicated to adjust. In addition, the MRD current driver is affected by its damper parameter changes and its own performance during operation, and the output accuracy and dynamic response of the control current cannot be guaranteed, which in turn reduces the damping control effect of the system, so it is necessary to design and study the driving system of the MRD. Therefore, this paper adopts the synchronous rectification BUCK circuit architecture, and uses the fuzzy PI control strategy to design a new digital current driver. It can have a strong adaptability to the parameter perturbation of the excitation coil of the damper while ensuring a fast response speed. The main work of this paper is as follows: (1) The parameter variation range of the MRD at different operating temperatures is analyzed experimentally, which can improve the parameter setting efficiency during the controller design process; (2) Deriving the mathematical model of the equivalent circuit after adding the load to provide a theoretical basis for the subsequent controller parameter tuning; (3) The fuzzy PI control strategy of the current driver of the MRD is established to improve the system stability in the face of the parameter changes and disturbances of the controlled object, and the robustness of the proposed control strategy is verified by experiments.

## 2. System Model Establishment and Analysis

### 2.1. Variation Analysis of MRD Coil Parameters

**Figure 1:**
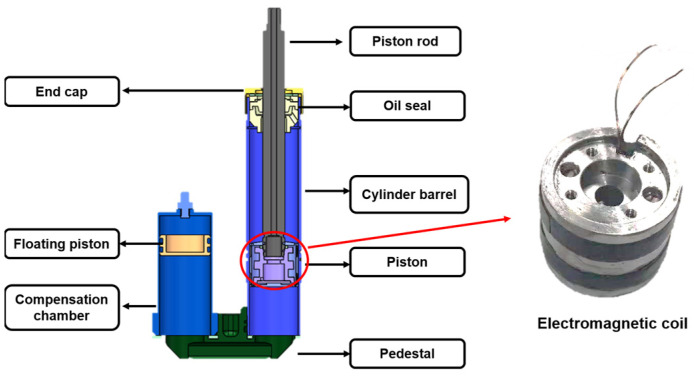
Magnetorheological damper and internal coil structure diagram.

**Figure 2:**
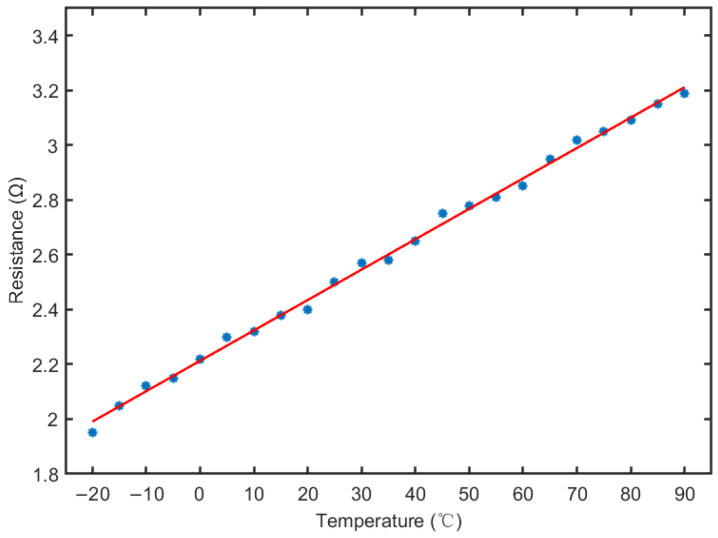
Resistance value and fitting curve of MRD at different working temperatures.

As shown in the Figure 1, the MRD designed by our team is mainly composed of pistons, cylinders, bases, floating pistons, and compensation air chambers. The excitation coil inside the damper can be modeled as the series connection of the resistance *R* and the inductance *L* [29]; the literature [16] pointed out that its internal resistance characteristic is affected by the temperature characteristic of the material, which changes linearly, and is given by

(1) $$R(T)=k_{R}T+R_{0}$$

where $k_{R}$ is the temperature coefficient of the coil material, *T* is the working temperature (°C) of the coil and $R_{0}$ is the equivalent resistance of the coil at 0 °C. As the MRD has an operating temperature range of −40 °C to 120 °C, the results of measuring the coil resistance (blue circles) at different temperatures are shown in Figure 2. After fitting using the least squares method, the relationship between resistance and temperature is obtained as shown in the red line in the figure, and the equation is shown in (2). the rate of change of the coil’s resistance at the highest and lowest operating temperatures of the MRD is obtained as 41.7% and −29.6%, respectively.

(2) R(T)=0.0111T+2.2128

### 2.2. Mathematical Model of Current Drive Circuit

**Figure 3:**
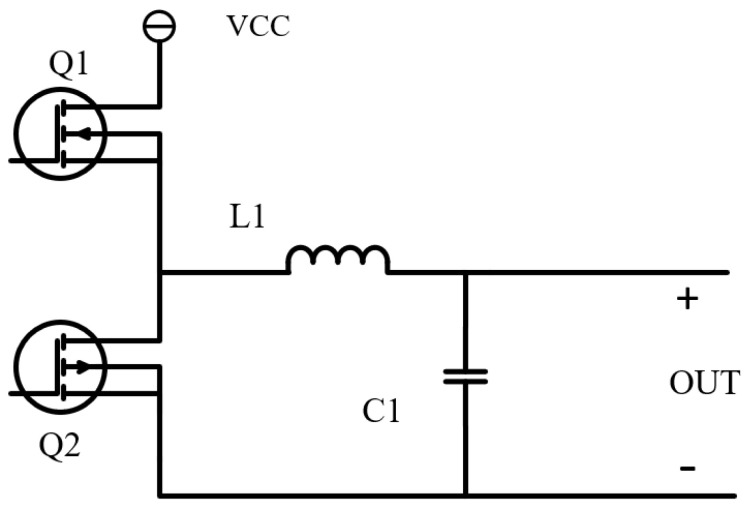
Synchronous rectification-type buck circuit structure diagram.

As shown in the Figure 3, the current driver design consists of a synchronous rectified buck circuit. Compared with the traditional step-down circuit, the MOS tube is used to replace the original rectifier diode. Since the internal resistance of the MOS tube is small, the conduction voltage drop and internal resistance loss of the circuit can be reduced [30], and the working efficiency of the circuit can be improved when it is used in a current output circuit.

**Figure 4:**
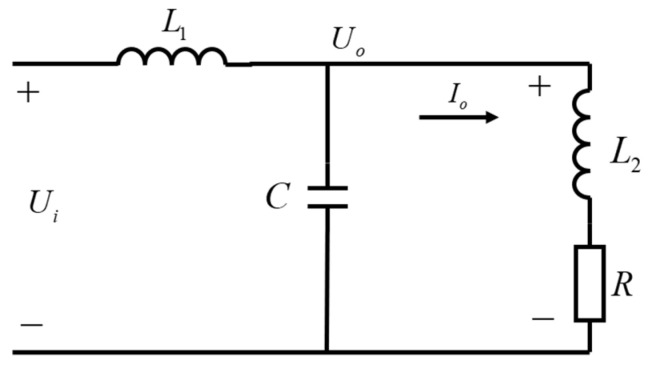
Equivalent circuit model added to load the main topology circuit switch model.

Based on the drive control loop composed of the existing buck drive circuit and the MRD circuit model, the equivalent mode of the switch model of the main topology circuit is shown in Figure 4. In order to provide theoretical support for the subsequent PI parameter tuning, the system equations of the drive circuit are established as follows, based on the principle of the time-averaged equivalence principle of the switching circuit [31].

(3) $$\left\{ \begin{array}{l} L_{1}\frac{di_{1}(t)}{dt}=u_{i}(t)-u_{O}(t)\\ u_{O}(t)=L_{2}\frac{di_{o}(t)}{dt}+Ri_{o}(t)\\ i_{C}(t)=C\frac{du_{o}(t)}{dt}\\ i_{i}(t)=i_{C}(t)+i_{o}(t) \end{array}\right.$$

where $u_{i}(t)$ is the output voltage of the input MOS tube, $L_{1}$ is the circuit inductance, C is the circuit capacitance, $i_{1}(t)$ is the current in $L_{1}$, $i_{C}(t)$ is the current in the capacitor C, $i_{o}(t)$ is the current in the damper loop, $L_{2}$ is the equivalent internal inductance of the damper and its value is 3.13 mH, R is the equivalent internal resistance of the damper and its value is 2.5 Ω, and $u_{O}(t)$ is the equivalent driver output voltage.

The transfer function of input voltage $u_{in}$ and output current $i_{o}$ is solved as:

(4) $$G(s)=\frac{I_{o}\left(s\right)}{U_{in}(s)}=\frac{1}{L_{1}L_{2}CS^{3}+L_{1}RCS^{2}+(L_{1}+L_{2})S+R}$$

### 2.3. Calculation of Drive Circuit Parameters

In order to reduce the circuit structure complexity and control difficulty of the current drive device, this paper selects the BTN8962TA integrated current half-bridge chip from Infineon (Neubiberg, Germany). The device includes a P-channel high-side MOSFET and an N-channel low-side MOSFET, eliminating the need for a charge pump. The chip integrates the drive logic unit and has the function of generating dead time internally. Only one PWM drive signal is needed to complete the driving of the half-bridge circuit. The peripheral circuit is simple and stable, which is suitable for the application environment of the current drive in this paper.

Let the input power input range be $V_{in}$, and the output voltage is $V_{out}$, the operating frequency is $F_{sw}$, and the rated output current is $I_{o}$; the calculation formula of the inductance value under critical conditions is expressed as

(5) $$I_{pk}=2I_{o}$$

(6) $$L=\frac{V_{out}}{I_{pk}}\frac{1}{F_{sw}}(1-\frac{V_{out}}{V_{in,\max}})$$

In the step-down circuit, whether it is the on state or the off state, the current change of the capacitor is always similar to that of the inductor [32], and the current change of the load after the capacitor is filtered and can be ignored; the formula for calculating the capacitance is:

(7) $$C=\frac{\Delta i_{o}}{8F_{sw}\Delta V_{out}}$$

where $\Delta i_{o}$ is the output current ripple and $\Delta V_{out}$ is the output voltage ripple.

In this paper, the DC 12 V power supply is selected, the rated current output is 3 A, the PWM switching frequency is 20 kHz, and the parameters are brought into Equations (6) and (7) to obtain the inductance value L = 4.7 × 10^−5^ H and capacitance value C = 3 × 10^−5^ F.

## 3. Controller Design

**Figure 5:**
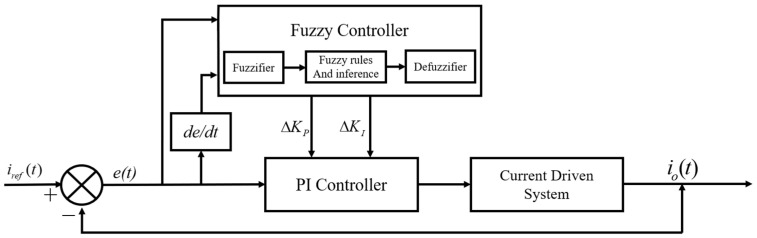
Fuzzy PI controller block diagram.

In order to overcome the influence of the system model change and disturbance, the fuzzy PI controller is used to control the system. The principle of fuzzy PI control is shown in Figure 5. The error e between the driver output current $i_{o}$ and the reference current $i_{ref}$ and its change rate ec are used as the input, and the PI parameters adjustment $\Delta K_{p}$ and $\Delta K_{i}$ are used as the output. According to the system transfer function and converter, the mathematical model determines the initial values $K_{p0}$ and $K_{i0}$ of the PI control.

### 3.1. PI Controller Design

The PI control principle is to use the proportional link and the integral link to form a linear combination of control variables, adjust the system deviation of the controlled object in real time [33], and make the output signal of the controlled object meet the calibration index. The continuous time domain PI mathematical expression is:

(8) $$u\left(t\right)=K_{p}\left(e\left(t\right)+\frac{1}{T_{i}}\int e\left(t\right)dt\right)$$

where e(t) is the control error, $K_{p}$ is the proportional constant, $T_{i}$ is the integral time constant, and the corresponding transfer function is:

(9) $$G_{s}=K_{p}\left(1+\frac{1}{T_{i}s}\right)$$

Because the current driver runs on a digital processing chip, a digital PI controller is required. The positional PI algorithm is to discretize the continuous time parameter t with the fixed sampling time T, and high static error control accuracy. Its mathematical expression is:

(10) $$u\left(k\right)=K_{p}\left(e\left(k\right)+\frac{T}{T_{i}}\sum_{i=0}^{k}e\left(i\right)\right)$$

where u(k) e(k) is the output and deviation of the controller in the kth sampling period, where $K_{i}=\frac{K_{p}T}{T_{i}}$ is used as the integral coefficient, and the function expression of the positional PI controller can be obtained:

(11) $$u\left(k\right)=K_{p}e\left(k\right)+K_{i}\sum_{i=0}^{k}e\left(i\right)$$

**Figure 6:**
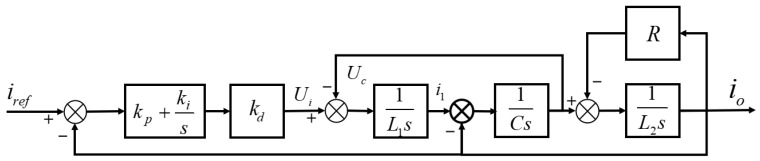
PI controller structure diagram.

According to the circuit equivalent model in Section 2, the transfer function block diagram of the PI controller of the system is shown in Figure 6.

It can be observed from Figure 6 that without considering the disturbance, its transfer function is:

(12) $$G_{i}(s)=\frac{K}{L_{1}L_{2}CS^{3}+L_{1}RCS^{2}+(L_{1}+L_{2})S+K}$$

In (12), the equivalent gain of the control object is $K=(K_{p}+K_{i}/s)/K_{d}$. In order to ensure that the corresponding phase at the crossover frequency is greater than −180°, the zero point of the PI controller is set at the crossover frequency. Let the switching frequency be $f_{sw}$. In order to better suppress the switching noise and improve the dynamic performance, the crossover frequency $f_{c}$ is selected as 1/10 of the switching frequency. At the crossover frequency, the system amplitude gain is 1, and the zero frequency of the PI regulator $f_{z}$ can be approximated as the crossover frequency. At this time, the PI controller parameters are obtained as:

(13) $$\{\begin{array}{c} \left|G_{i}(j2\pi f_{c})\right|=1\\ \frac{K_{I}}{K_{p}}=2\pi f_{z} \end{array}$$

Since the on-resistance, parasitic capacitance, and inductance components of the components are not considered in the system modeling, the model has certain uncertainty. The PI parameters derived from the transfer function cannot be guaranteed to be the global optimal solution.

### 3.2. Fuzzy PI Controller Design

In order to overcome the influence of the system model change and disturbance, the fuzzy PI controller is used to control the system. The principle of the fuzzy PI control is shown in Figure 5. The error e between the driver output current $i_{o}$ and the reference current $i_{ref}$ and its change rate ec are used as the input, and the PI parameters’ adjustment $\Delta K_{p}$ and $\Delta K_{i}$ are used as the output.

(14) e(t)=iref(t)−io(t)ec=de(t)dt

According to the system transfer function and converter, the mathematical model determines the initial values $K_{p0}$ and $K_{i0}$ of the PI control. For a fuzzy controller, the more linguistic variables are selected, the more accurate the control system will be, but it will increase the complexity of the system, bring more computation and affect the real-time performance of the system. Therefore, in this paper, after balancing the complexity of the controller and the accuracy of the system, seven fuzzy subsets {NB, NM, NS, ZE, PS, PM, and PB} are used to assign the input linguistic variables, representing {negative large, negative medium, negative small, zero, positive big, positive middle, and positive small}, reflecting the magnitude and direction of the input and output deviation, and representing {negative large, negative medium, negative small, zero, positive big, positive middle, and positive small}, reflecting the magnitude and direction of input and output deviation. The discourse domain of the inputs is set as [−6, 6]. Hence, the input variables need to be scaled in such a manner that their minima are −6 and maxima are 6.

(15) $$\begin{array}{l} k_{e}=\frac{E_{\max}}{e_{\max}}\\ k_{ec}=\frac{EC_{\max}}{ec_{\max}} \end{array}$$

where $k_{e}$ and $k_{ec}$ represent the quantification factor of the error and the one of the error’s change, respectively, $e_{\max}$ and $ec_{\max}$ are the maxima of the input variables e and ec, respectively; and $E_{\max}$, $EC_{\max}$ are the maxima of the inputs’ discourse domain, which are six in the article.

**Figure 7:**
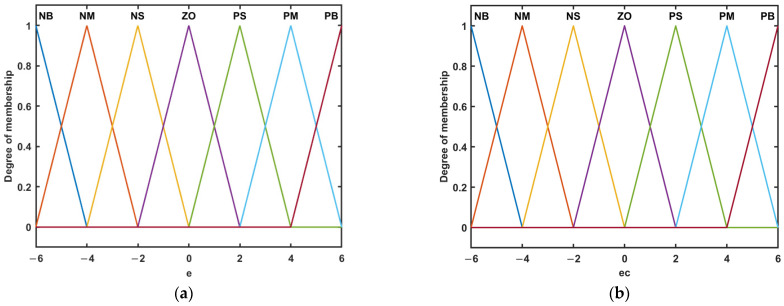
Trigonometric membership functions: (**a**) *e*; (**b**) *ec*.

Fuzzifier. Different membership function shapes affect the comprehensive performance of the fuzzy controller [34]. Since the current driver system is a complex nonlinear system, the parasitic loss in its circuit topology and the influence of the external environment will cause the system to fluctuate. Therefore, we need a membership function, which is sensitive to these fluctuations. When the input deviation changes, the triangular membership function is more sensitive than other shapes [35]; its mathematical expression is:

(16) $$f(x,a,b,c)=\{\begin{array}{cc} 0 & x\leq a\\ \frac{x-a}{b-a} & a\leq x\leq b\\ \frac{c-x}{c-b} & b\leq x\leq c\\ 0 & x\geq c \end{array}$$

where a and c represent the two base points of the triangle corresponding to a degree of the membership of 0, and b represents the midpoint of the triangle, which corresponds to the maximum degree of membership; in this paper, the affiliation functions of e and ec are shown in Figure 7.

**Figure 8:**
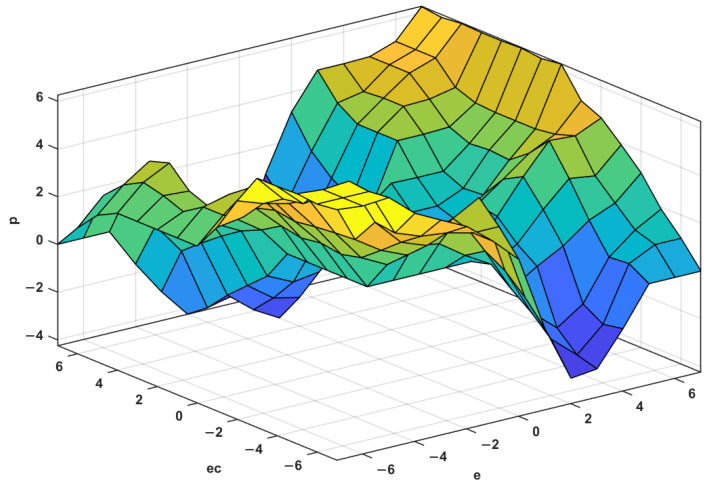
Spatial distribution of Δ*Kp*.

**Figure 9:**
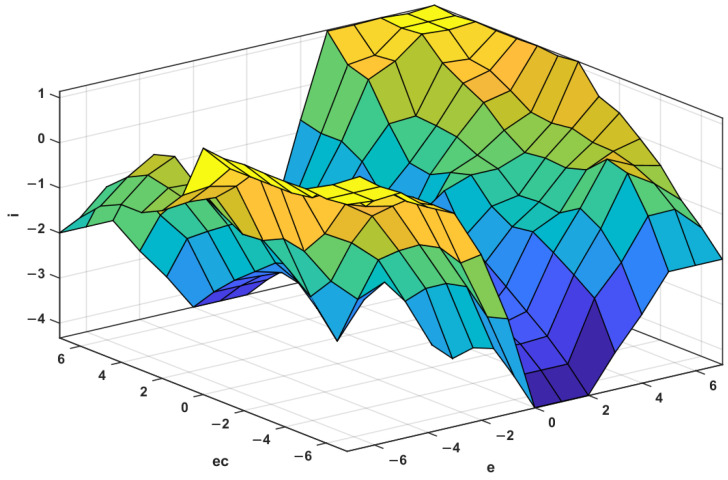
Spatial distribution of Δ*Ki*.

Fuzzy rules and fuzzy inference. For the proportional control term $K_{p}$, its role is to proportionally reflect the error signal e of the control system. When the error occurs, the controller immediately generates a control function to reduce the error. Excessive $K_{p}$ will cause the system to oscillate and destroy the dynamic performance of the system. Therefore, the $K_{p}$ value needs to be selected correctly. When e is large, $K_{p}$ takes a large value to improve the response speed; meanwhile, when e is small, $K_{p}$ decreases to prevent the overshoot from causing the oscillation to stabilize the system as soon as possible. At the same time, it should also be considered that when ec and e have the same sign, the output deviates from the stable value, and $K_{p}$ should be appropriately increased, otherwise, $K_{p}$ should be appropriately reduced. Similarly, the adjustment method of the integral control term $K_{i}$ is similar to that of $K_{p}$. Finally, based on the engineering control experience, the fuzzy control and adjustment rules for $\Delta K_{p}$ and $\Delta K_{i}$ are established according to the variation rules of e and ec. The spatial distribution and tables of the fuzzy rules are shown in Figure 8 and Figure 9, and Table 2 and Table 3, respectively.

The input of the fuzzy controller studied in this paper is the voltage deviation and deviation variation. When the deviation E of the given voltage and the deviation change EC, the control output of the controller is U, and the Mamdani method [36] is selected as the fuzzy logic inference method. The Mamdani inference method uses the Cartesian product of A and B to represent the fuzzy from A to B.

(17) $$if{\;E=A}_{i}\;and\;EC{=B}_{i},\;then\;U{=C}_{i},\;\;\;i=1,2,\ldots 49$$

$A_{i}$ represents the corresponding fuzzy subset on the universe U,$B_{i}$ represents the corresponding fuzzy subset on the universe V, and the control decision table R can be formulated according to expert experience; where R represents the corresponding fuzzy subset on the Cartesian product set U×V, the output control quantity can be expressed as:

(18) C=(A⋅B)∘R

where A⋅B is the direct product of the fuzzy sets A and B, and C is the synthesis of the former and the fuzzy matrix R; thus, the values of $\Delta K_{p}$ and $\Delta K_{i}$ can be calculated respectively, according to the above formula.

Defuzzifier. In order to obtain an accurate control quantity, the fuzzy method is required to express the calculation result of the output membership function well. The gravity method [37] uses the center of gravity of the region enclosed by the affiliation function curve and the horizontal coordinates as the final output value of the fuzzy inference, which has a smoother output inference control. The output changes even in response to small changes in the input signal. For the discrete threshold case with *m* output quantization levels, it is assumed that the membership function corresponding to the fuzzy set $v_{k}$ on the universe $v_{k}$ is $u_{k}(v_{k})$, and the abscissa of the center of gravity is $v_{o}$, which is expressed as

(19) $$v_{o}=\frac{\sum_{k=1}^{m}v_{k}\mu_{v}(v_{k})}{\sum_{k=1}^{m}\mu_{v}(v_{k})}$$

The fuzzy set distribution is determined by selecting the input voltage deviation and deviation change, and then the fuzzy logic inference is carried out through the fuzzy rule table, and the adjustment values $\Delta K_{p}$ and $\Delta K_{i}$ of the PI control parameters are output. Using the center of gravity method for defuzzification can obtain the clear control parameter value, superimpose it with the initial set value of the PI controller, and obtain the final set value of the fuzzy PI control parameter:

(20) $$\begin{array}{l} K_{p}=K_{p0}+\Delta K_{p}\\ K_{i}=K_{i0}+\Delta K_{i} \end{array}$$

## 4. Experiment and Discussion

**Figure 10:**
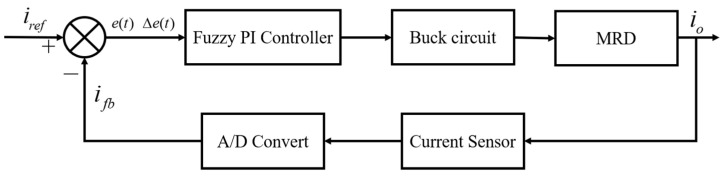
Block diagram of current driver.

In order to verify the effectiveness of the controller, the dynamic and steady state performance tests of the current driver are carried out in this section. The block diagram of the current drive control system is shown in Figure 10. The system mainly consists of MRD, current sampling digital to analogue converter, communication module, and synchronous rectifier power output.

**Figure 11:**
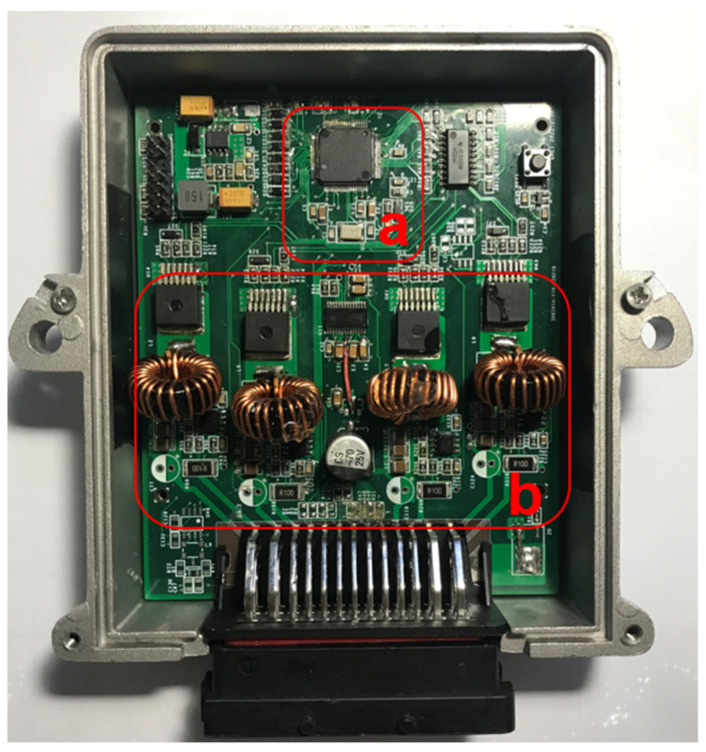
Current driver physical diagram: (**a**) DSP core; (**b**) 4-channel current output circuit.

As shown in Figure 11, the current driver DSP core adopts the TMS320F28035 digital signal processing chip of TI (Texas Instruments). DSP calculates the error between the current feedback value and the set value, and adjusts the difference in real time through the control law, so that the current output value is infinitely close to the set value, and the closed-loop control of the output current is realized.

**Figure 12:**
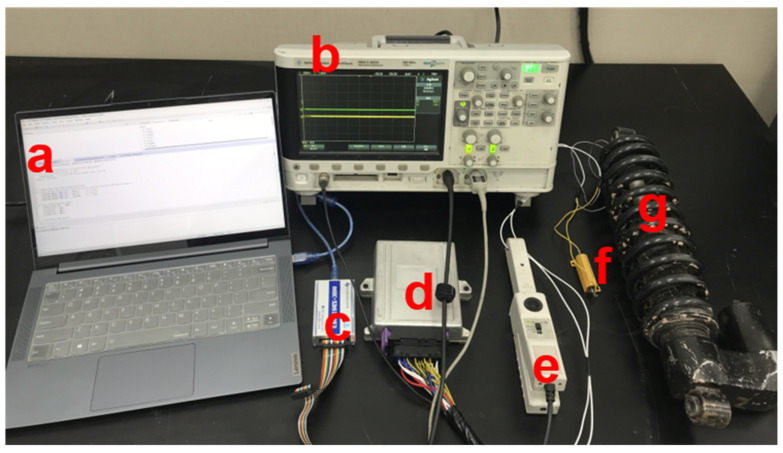
Schematic diagram of current driver test: (**a**) PC; (**b**) oscilloscope/signal generator; (**c**) JTAG; (**d**) current driver; (**e**) current probe; (**f**) load resistor 1 Ω; (**g**) MRD.

**Figure 13:**
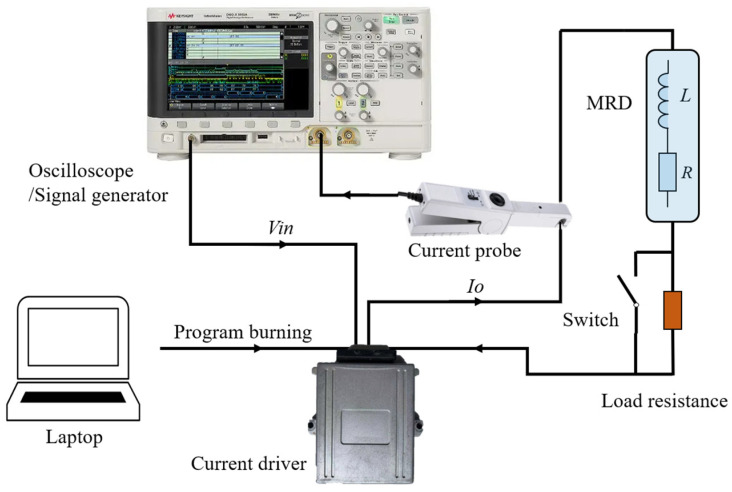
Current driver test connection block diagram.

The current output control object of this paper is the MRD designed and developed by our group. The test block diagram is shown in Figure 12; it is mainly composed of an oscilloscope/signal generator, current driver, current probe, MRD, load resistor, and PC. The working principle of the system is shown in Figure 13, where the signal generator generates a current control level signal and transmits it to the driver, and the driver converts the control signal into the driving current of the MRD under the action of the controller. By adding a load resistance at a specific time, the parameter changes of the MRD are simulated, and the output signal of the driver is collected through a current probe to detect the start-up response and dynamic adjustment performance of the current driver under different control algorithms.

### 4.1. Current Driver Startup Response Performance Test

#### 4.1.1. Drive Dynamic Response under Fixed Control Object Parameters

At normal temperature, when the load is an MRD, the current driver applies a step current of 1A, 2A, and 3A to the load under the PI controller and fuzzy PI controller, respectively, to test the output response of the driver under different algorithms.

#### 4.1.2. Dynamic Performance Test of Current Driver after Parameter Change of Control Object

Under natural temperature conditions, a 1Ω load resistance is connected to the load loop of the magnetorheological damper, and the internal resistance of the magnetorheological damper changes by 40%. In this case, the PI controller and the fuzzy PI controller are used, respectively. Step currents of 1A, 2A, and 3A are applied to the load to detect the start-up response performance of the driver under different control laws.

**Figure 14:**
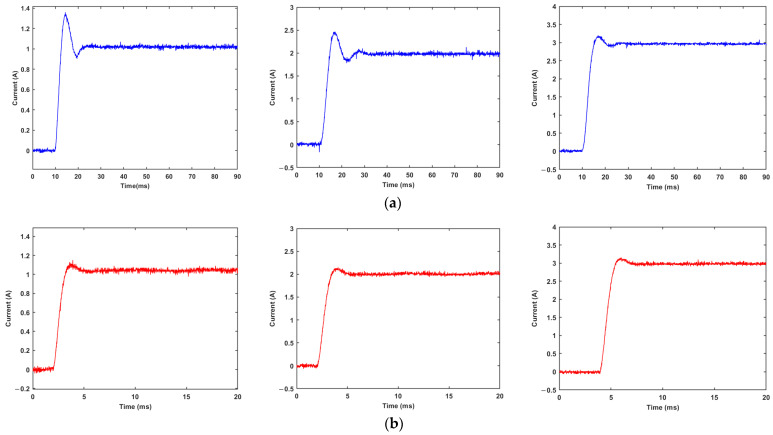
Response of current driver at 1 A, 2 A, 3 A step current: (**a**) PI controller; (**b**) fuzzy PI controller.

According to the experimental results in Figure 14 and Table 4 and Table 5, when the parameters are fixed, the fuzzy PI controller exhibits a faster steady-state response time, which can save up to 50% of the tuning time compared to the PI controller. In terms of overshoot, the overshoot of the PI controller reaches 34% when the output current is small, while the overshoot of the fuzzy PI controller is controlled within 10% under different output current conditions. It demonstrates that the fuzzy PI controller can guarantee the output accuracy and maintain a high dynamic response under different current outputs.

**Figure 15:**
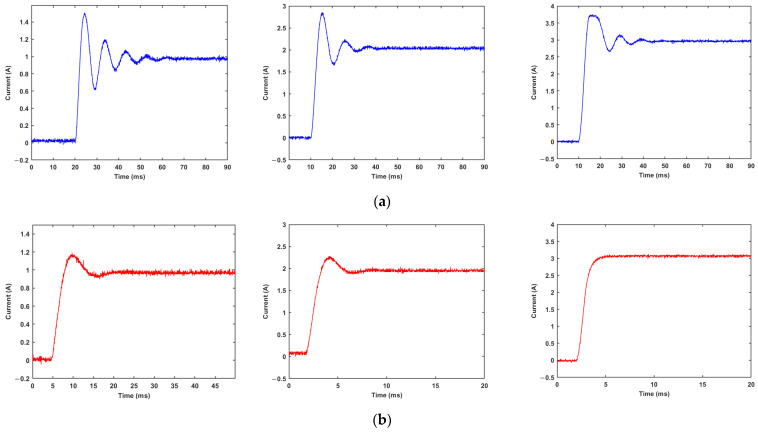
Response of current driver at 1 A, 2 A, 3 A step current after parameter changes: (**a**) PI controller; (**b**) fuzzy PI controller.

From the experimental results in Figure 15 and Table 6 and Table 7, after the load parameters are changed, the PI control performance is seriously degraded, and the output current of the driver oscillates. The deterioration is more serious at a low current output, the overshoot can reach 47% at most, and the adjustment time also increases to more than 30 ms, indicating that the PI controller has become unstable at this time. However, the adjustment time of the fuzzy PI controller is only increased by 3 ms, and the overshoot is also well controlled, indicating that the fuzzy PI controller has stronger robustness to the change of load parameters.

### 4.2. Control Object Parameter Disturbance Current Driver Performance Test

Due to the complex environment of the MRD application, its working conditions are complicated and changeable, which requires the controller to have a strong dynamic adjustment capability. Therefore, at a natural temperature, when the driver is connected to the MRD and the output current is in a stable state connected to the load reset resistor. Simulate the dynamic adjustment performance of the PI controller and fuzzy PI controller when the parameters of the controlled object are disturbed, respectively.

**Figure 16:**
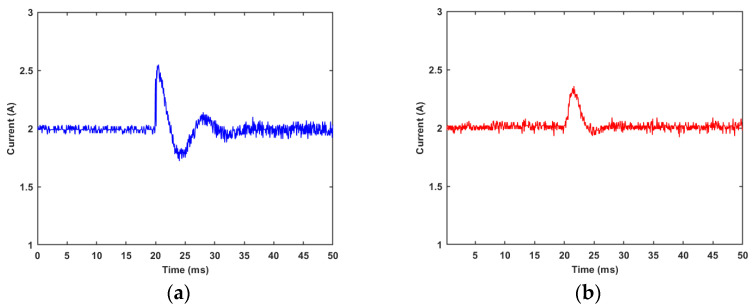
Responses of different controllers under the disturbance of damper parameters (2 A): (**a**) PI controller; (**b**) fuzzy PI controller.

It can be observed from Figure 16 that when the MRD parameters are disturbed, the adjustment time of the fuzzy PI controller is about 4.7 ms, while the PI controller needs about 7.0 ms. Furthermore, the overshoot of the latter also exceeds the former. It shows that the dynamic adjustment performance of the fuzzy PI controller to the MRD is better than that of the traditional PI controller.

From the analysis above, it can be concluded that: (1) when MRD parameters are fixed, the overshoot of the PI controller is more serious at a low current output. The reason is that the circuit parameters and PI parameters are obtained in the rated current output state when the circuit parameters and PI parameters are set and calculated, and the fixed PI parameters are obtained at different current output. However, the global optimal performance cannot be guaranteed. The fuzzy PI controller has better response characteristics, reduces the adjustment time and overshoot of the system, and ensures the comprehensive performance of the system under different current output states. (2) When the MRD parameter changes by 40%, the PI controller is unstable due to the fixed PI parameter. The fuzzy PI controller demonstrates strong robustness. Although the control performance is reduced, the accuracy of the current output is still guaranteed. In addition, when the load is disturbed under the steady state output of the system, the fuzzy PI controller is also better than the PI controller in the control of the adjustment time and overshoot, and has a strong dynamic adjustment ability.

To sum up, due to the application background of MRD, due to the change of the parameters of the driver facing the load and the different current output states, the output needs to be adjusted quickly and accurately. The parameters of the PI controller are fixed, which cannot guarantee the global optimal control effect and affect the control accuracy. Compared with the PI controller, the fuzzy PI controller improves the response speed while reducing the system overshoot, shows stronger robustness, and can effectively improve the performance of the current drive.

## 5. Conclusions

In this paper, we address the problem that the internal resistance value of MRD may change during operation due to the influence of temperature, which leads to the poor current output response of the driver. First, by experientially analyzing the parameter variation range of MRD at different operating temperatures, the mathematical model of the synchronous rectifier BUCK circuit is established and the circuit component parameters are solved, and the hardware platform design of the driver is completed. Then, a magnetorheological damper current driving system based on the fuzzy PI control is designed. Experiments demonstrate that the magnetorheological internal parameters vary by 40%, compared with the fixed-parameter PI controller; meanwhile, the fuzzy PI controller shows better parameter adaptation capability, which can significantly reduce the adjustment time and overshoot of the output current. When the system is disturbed, the dynamic adjustment performance of the fuzzy PI controller is stronger, which indicates that the designed fuzzy PI controller has higher robustness in the face of the change in the control focal parameters.

## Figures and Tables

**Table 1:** Performance comparison between different controllers.

Controller	Advantages	Disadvantages
PI	Simple structure, high reliability, and real-time	Difficult parameter adjustment; poor robustness
Fuzzy PI	Less calculation, strong robustness, and high real-time performance	difficult parameter adjustment
SMC	Fast response, strong robustness, and simple structure	Low precision; controller chattering
ADRC	Strong robustness, model independent, and high precision	Large amount of calculation, complex parameters, and poor real-time performance

**Table 2:** Δ*Kp* fuzzy inference rule table.

e	ec						
	NB	NM	NS	ZO	PS	PM	PB
NB	PB	PB	PB	PB	PS	PS	ZO
NM	PB	PB	PM	PM	PS	PM	ZO
NS	PM	PS	ZO	ZO	ZO	NS	NM
ZO	PS	PM	ZO	ZO	ZO	NM	NS
PS	NM	NS	ZO	ZO	ZO	PS	PM
PM	ZO	ZO	PM	PS	PS	PM	PM
PB	ZO	PS	PM	PB	PB	PB	PB

**Table 3:** Δ*Ki* fuzzy inference rule table.

e	ec						
	NB	NM	NS	ZO	PS	PM	PB
NB	PB	PB	PB	PB	PB	PS	ZO
NM	PB	PB	PM	PM	PS	PM	ZO
NS	PM	PM	PS	NM	ZO	NS	NM
ZO	NM	NS	NM	ZO	NM	NS	NM
PS	NM	NS	ZO	PS	PS	PM	PB
PM	ZO	PM	PS	PS	PM	PB	PB
PB	ZO	PS	PM	PB	PB	PB	PB

**Table 4:** Adjustment time of PI and fuzzy PI controllers at different step currents.

Response Parameter	Current (A)	PI	Fuzzy PI
Adjustment time	1	12.5 ms	7 ms
	2	15 ms	7.5 ms
	3	15 ms	8 ms

**Table 5:** Overshoot of PI and fuzzy PI controllers at different step currents.

Response Parameter	Current (A)	PI	Fuzzy PI
Overshoot	1	34%	9%
	2	20%	6%
	3	8%	5.3%

**Table 6:** Adjustment time of PI and fuzzy PI controllers at different step currents after parameter changes.

Response Parameter	Current (A)	PI	Fuzzy PI
Adjustment time	1	30 ms	9 ms
	2	32 ms	11 ms
	3	32 ms	7 ms

**Table 7:** Overshoot of PI and fuzzy PI controllers at different step currents after parameter changes.

Response Parameter	Current (A)	PI	Fuzzy PI
Overshoot	1	47%	17%
	2	40%	12.5%
	3	23.3%	--

## Data Availability

Not applicable.
